# Depression after Infection with West Nile Virus^1^

**DOI:** 10.3201/eid1303.060602

**Published:** 2007-03

**Authors:** Kristy O. Murray, Melissa Resnick, Vicki Miller

**Affiliations:** * University of Texas Health Science Center, Houston, Texas, USA

**Keywords:** West Nile virus, encephalitis, depression, personality changes, CES-D scale, clinical sequelae, dispatch

## Abstract

Previous reports have noted depression after West Nile virus (WNV) infection. We further measured this outcome and found that 31% of patients reported new-onset depression and 75% of these had Center for Epidemiologic Studies Depression scores indicative of mild-to-severe depression. Physicians should be aware of neuropsychiatric consequences of WNV in patients.

West Nile virus (WNV) was identified for the first time in the Western Hemisphere in New York City in 1999; since then, a dramatic westward and southward spread of WNV activity has occurred in the United States (*1*,*2*). In 2002, WNV was identified in the Houston, Texas, metropolitan area, resulting in 105 human cases (*3*).

Long-term clinical sequelae after infection are still being defined. A year after the outbreak of WNV in New York City, 38% of patients subjectively reported depression (*4*). Another 1-year follow-up in Colorado noted that 23% of patients reported anxiety and depression (*5*). In Houston, we have been conducting a prospective study that involves both subjective and objective measurements of physical, neurologic, and cognitive functioning of patients identified with symptomatic WNV infections. We describe the subjective and objective evaluations of depression in these patients.

## The Study

Clinical WNV patients, confirmed by immunoglobulin M ELISA and identified through local surveillance in the Houston metropolitan area from 2002 through 2004, were invited to participate in a study to determine long-term clinical sequelae. Those consenting to participate were interviewed. No patient was denied participation on the basis of age, sex, race, or ethnicity. Excluded patients included those who were residing outside of the Houston area, deceased, or lost to follow-up. This study was reviewed and approved by the University of Texas Health Science Center Institutional Review Board (HSC-SPH-03–039).

Initial interviews were conducted after hospitalization, and follow-up interviews were conducted every 6 months until the patient reported being back to pre-WNV infection functioning. For the 1-year follow-up, interviews were conducted during each December at the end of transmission season. Interviews were mainly conducted over the telephone, but a small proportion were conducted in person. In all of these interviews, a higher than expected proportion of patients reported depression immediately after their illness. To better assess this quantitatively, we incorporated the Center for Epidemiologic Studies Depression (CES-D) scale (*6*) into both initial and follow-up interviews. This scale is a commonly used method for objective measurement of clinical depression. The tool is composed of 20 questions focused on self-report of depressive moods and behavioral changes experienced by the patient during 1 week. The resulting scores were interpreted as follows: 1) <15, the patient is not experiencing depression; 2) 15–21, the patient may be experiencing mild-to-moderate depression; 3) >22–60, the patient may be experiencing major depression. In addition to the objective measurement using the CES-D scale, we also asked patients if they were experiencing depression since their illness and if they had a previous history of depression, with yes/no responses elicited. Barthel Index scores were used to quantitatively evaluate level of physical functioning and disability in patients; a score of 100 points indicated no physical disability. Because patients also commonly reported a change in personality immediately after WNV infection, we assessed this finding subjectively and asked those reporting a change to describe what they were experiencing.

Data were analyzed by using NCSS statistical software (Kaysville, UT, USA). With the Kruskal-Wallis 1-way analysis of variance on ranks, we analyzed CES-D scores and WNV outcome; CES-D scores and sex, age, and depression (CES-D score of >15); and physical functioning (Barthel Index) and depression.

A total of 65 patients were interviewed; 38 (58%) cases had encephalitis when initially evaluated, 19 (29%) had meningitis, and 8 (12%) had fever. The mean age of patients was 55 years (range 12–86 years). Most patients were white, non-Hispanic (80%), followed by black (11%) and white, Hispanic (9%).

One year after infection with WNV, 26 patients reported experiencing depression. Of these, 6 reported a history of depression before infection. Of the 20 patients considered to have new-onset depression, 13 (65%) had a clinical diagnosis of WNV encephalitis (Table), and 10 (50%) were male. The mean CES-D scores for those who reported no depression was 5.5 (range 0–19) compared with a mean score of 22 (range 0–44) for those who reported depression. There was no statistical difference in CES-D scores between those who had encephalitis and those who had meningitis or fever (p = 0.19) or between those with West Nile neuroinvasive disease (encephalitis or meningitis) compared with those with fever (p = 0.55). On the basis of CES-D scores for those self-reporting depression since their illness with WNV, 5 (25%) patients were classified by CES-D as not depressed, 6 (30%) were classified as having mild-to-moderate depression, and 9 (45%) were classified as having major depression. Of the 39 patients who self-reported that they had not had depression since their WNV illness, 4 had a CES-D score of >15. No statistically significant associations were found between loss of physical functioning (Barthel index scores <100; p = 0.39), sex (p = 0.89), or age (p = 0.47) and depression (CES-D scores of >15).

**Table:** Subjective and objective measurements of depression and personality changes 1 year after clinical illness from WNV infection*

	All patients (%)	West Nile encephalitis		West Nile meningitis/ West Nile fever	
		Male (%)	Female (%)	Male (%)	Female (%)
Measurements					
New onset depression since WNV	20/65 (31)	8/27 (30)	5/11 (45)	2/12 (17)	5/15 (33)
Mean CES-D score for those reporting depression (range)	22 (0–44)	17 (7–30)	23 (0–37)	38 (32–43)	22 (0–44)
CES-D score ≥15	15/20 (75)	5/8 (63)	4/5 (80)	2/2 (100)	4/5 (80)
Taking antidepressants	7/20 (35)	3/8 (38)	1/5 (20)	1/2 (50)	2/5 (40)
Report antidepressants helping with symptoms	4/7 (57)	2/3 (67)	1/1 (100)	0/1 (0)	1/2 (50)
Receiving counseling	2/20 (10)	0/8 (0)	1/5 (20)	1/2 (50)	0/5 (0)
Report counseling helping with symptoms	2/2 (100)	0	1/1 (100)	1/1 (100)	0
Personality change since WNV	29/65 (45)	12/27 (44)	6/11 (55)	4/12 (33)	7/15 (47)
Anger/irritability/aggression	19/29 (66)	8/12 (67)	4/6 (67)	3/4 (75)	4/7 (57)
Decreased socialization	8/29 (28)	3/12 (25)	1/6 (17)	1/4 (25)	3/7 (43)
Increased sensitivity/cries easily	3/29 (10)	0/12 (0)	1/6 (17)	0/4 (0)	2/7 (29)
Feelings of hopelessness	1/29 (3)	1/12 (8)	0/6 (0)	0/4 (0)	0/7 (0)

*WNV, West Nile virus; CES-D, Center for Epidemiological Studies Depression.

Seven (35%) patients reported taking antidepressants for their symptoms; 4 of these patients reported at least some improvement. CES-D scores of 6 of these 7 patients were >15; 4 showed evidence of major depression. Two patients reported seeing a counselor for depression, including 1 who was also taking antidepressants. Both patients reported that the counseling was helpful; however, based on their CES-D scores, both were classified as having major depression at the time of the interview.

Personality changes were reported subjectively among 29 (45%) of the 65 patients (Table); 18 (62%) of these were in patients who had encephalitis, and 16 (55%) of these patients were male. When asked to describe their personality change, 19 patients (66%) reported an increase in anger (being short-tempered and irritable); 8 patients (28%) reported being less social; 3 patients (10%) reported increased sensitivity; and 1 patient (3%) reported feelings of hopelessness.

## Conclusions

Depression and personality changes after WNV infection have been briefly observed in previous studies (*4*,*5*); however, this study is the first known to objectively evaluate this outcome. As evidenced by subjective and objective measurements, depression is an important outcome in patients with a clinical diagnosis of WNV infection: indeed, 75% of those reporting no previous history of depression had high CES-D scores. Understanding the pathology of this outcome and determining whether the depression is situational (a result of prolonged recovery) or caused by chemical changes in the brain related to inflammation are critical. Depression was not associated with loss of physical functioning, sex, or age.

Depression after encephalitis, regardless of etiology, is not uncommon. Depression and personality changes in humans have been previously reported as a neuropsychiatric consequence of Lyme disease, Nipah virus, tickborne encephalitis virus, and Saint Louis encephalitis virus infections (*7*–*10*). After the encephalitis lethargica pandemic from 1917 to 1926, depression, mania, and obsessive-compulsive disorder were observed in postencephalitis patients (*11*). These observations led to the understanding of the role of the basal ganglia in mood, personality, and obsessional syndromes. In a mouse model experiment, infection of the brain with Venezuelan equine encephalitis virus resulted in a serotonin presynaptic deficit and postsynaptic hyperreactivity of the serotonin system (*12*).

Inflammation of the brain can result in an alteration in the neurotransmitter serotonin, which may lead to the development of mood disorders (*13*). Capuron et al. identified a significant proportion of patients in whom depression developed after cytokine therapy for cancer. The neurotoxic inflammation induced by cytokines resulted in decreased levels of tryptophan, the amino acid precursor for serotonin. This decrease was positively correlated with the development and severity of depressive symptoms in patients.

The long-term clinical sequelae of WNV neuroinvasive disease need to be further defined. By understanding potential outcomes and determining whether certain interventions such as physical therapy, counseling, and antidepressant drug therapy can improve recovery, we can better understand prognosis and potential treatment interventions. Physicians should note that depression and personality changes could be important neuropsychiatric consequences in patients with clinical WNV infection.

## References

[1] Nash D, Mostashari F, Fine A Miller J, O’Leary D, Murray K, et al. The outbreak of West Nile virus infection in the New York City area in 1999. N Engl J Med. 2001;344:1807–14. 10.1056/NEJM200106143442401 11407341

[2] Centers for Disease Control and Prevention. Assessing the capacity for surveillance, prevention, and control of West Nile virus infection—United States, 1999 and 2004. MMWR Morb Mortal Wkly Rep. 2006;55:150–3. 16484978

[3] Lillibridge KM, Parsons R, Randle Y, Travassos da Rosa APA, Guzman H, Siirin M, The 2002 introduction of West Nile virus into Harris County, Texas, an area historically endemic for St. Louis encephalitis. Am J Trop Med Hyg. 2004;70:676–81. 15211013

[4] Klee AL, Maldin B, Edwin B, Poshni I, Mostashari F, Fine A, Long-term prognosis for clinical West Nile virus infection. Emerg Infect Dis. 2004;10:1405–11. 15496241 10.3201/eid1008.030879 PMC3320418

[5] Sejvar JJ, Bode AV, Marfin AA, Campbell GL, Pape J, Biggerstaff BJ, West Nile virus–associated flaccid paralysis outcome. Emerg Infect Dis. 2006;12:514–6. 16704798 10.3201/eid1203.050643 PMC3291435

[6] Radloff LS. The CES-D Scale: a self-report depression scale for research in the general population. Appl Psychol Meas. 1977;1:385–401. 10.1177/014662167700100306

[7] Fallon BA, Nields JA. Lyme disease: a neuropsychiatric illness. Am J Psychiatry. 1994;151:1571–83. 7943444 10.1176/ajp.151.11.1571

[8] Ng BY, Lim CC, Yeoh A, Lee WL. Neuropsychiatric sequlae of Nipah virus encephalitis. J Neuropsychiatry Clin Neurosci. 2004;16:500–4. 10.1176/appi.neuropsych.16.4.500 15616178

[9] Schmolck H, Maritz E, Kletzin I, Korinthenburg R. Neurologic, neuropsychologic, and electroencephalographic findings after European tick-borne encephalitis in children. J Child Neurol. 2005;20:500–8. 15996399 10.1177/088307380502000606

[10] Greve KW, Houston RJ, Adams D, Stanford MS, Bianchini KJ, Clancy A, et al. The neurobehavioural consequences of St. Louis encephalitis infection. Brain Inj. 2002;16:917–27. 10.1080/02699050210131920 12419004

[11] Cheyette SR, Cummings JL. Encephalitis lethargica: lessons for contemporary neuropsychiatry. J Neuropsychiatry Clin Neurosci. 1995;7:125–34. 7626955 10.1176/jnp.7.2.125

[12] Lima L, Drujan B, Walder R. Cerebral serotonin in viral encephalitis. J Neural Transm Suppl. 1990;29:141–51. 2358799 10.1007/978-3-7091-9050-0_14

[13] Capuron L, Ravaud A, Neveu PJ, Miller AH, Maes M, Dantzer R. Association between decreased serum tryptophan concentrations and depressive symptoms in cancer patients undergoing cytokine therapy. Mol Psychiatry. 2002;7:468–73. 10.1038/sj.mp.4000995 12082564

